# Seasonal and sex-related variation in vitamin D status and its association with other biochemical markers in young individuals: A cross-sectional study

**DOI:** 10.1371/journal.pone.0298862

**Published:** 2024-03-29

**Authors:** Marcela Moraes Mendes, Maísa Miranda Araújo, Patrícia Borges Botelho, Kênia Mara Baiocchi de Carvalho

**Affiliations:** 1 Department of Nutrition, Graduate Program in Human Nutrition, University of Brasilia, Brasília, Federal District, Brazil; 2 Postgraduate Program, Faculty of Nutrition, Federal University of Goias, Goiania/GO, Brazil; Endocrinology and Metabolism Population Sciences Institute, Tehran University of Medical Sciences, ISLAMIC REPUBLIC OF IRAN

## Abstract

**Background:**

While several studies have investigated the association between vitamin D deficiency and biochemical parameters, the results are still inconsistent and mostly overlook seasonal variations. This study explored the relationships between 25-hydroxy-vitamin D (25(OH)D) concentrations, biochemical markers, and seasonal variation among young males and females.

**Methods:**

A cross-sectional study was conducted among 203 individuals aged 18–24 years of both sexes residing in Brasilia, Brazil (latitude: 15°S). Sociodemographic variables, season of blood collection, and serum levels of 25(OH)D, total cholesterol, HDL-cholesterol, LDL-cholesterol, triglycerides, glycated hemoglobin (HbA1c), glucose, insulin, hs-CRP, parathyroid hormone, ionized calcium, and alkaline phosphatase were included. Descriptive statistics and differences among groups, correlations, and linear regression tests were performed.

**Results:**

The mean age of the participants was 21.17±1.7 years, and the mean serum 25(OH)D level was 25.76±7.0 ng/mL. Of the participants, 50.7% had vitamin D insufficiency (20 to 29.9 ng/mL), and 23.2% were vitamin D deficient (≤20 ng/mL). Vitamin D deficiency was higher in the spring (53.2%) and among females (29.5%). In young men with vitamin D insufficiency/deficiency (≤29.9 ng/mL) (n = 49), 25(OH)D levels were inversely correlated with HOMA-β (r = -0.234, p = 0.032) and triglyceride (r = -0.415, p = 0.003) levels. However, there were no significant correlations between 25(OH)D concentrations and biochemical markers among women with insufficient and deficient vitamin D levels.

**Conclusion:**

This study found a high prevalence of vitamin D insufficiency/deficiency among young individuals living in Brasília, Brazil, particularly women and during the spring season. Our findings suggest that lower 25(OH)D levels (≤29.9 ng/mL) may be associated with insulin resistance and an increased risk of cardiovascular disease in young men studied. However, further studies with larger representative samples are needed to explore the mechanisms underlying the association between vitamin D and biochemical parameters.

## Introduction

Vitamin D is primarily obtained through endogenous synthesis (80–90%), which occurs via ultraviolet B (UVB) radiation on the epidermis, as well as from dietary sources [1]. Given the critical role of sunlight exposure in maintaining vitamin D levels, it was previously assumed that hypovitaminosis D would not be a concern in populations residing in lower latitude countries, where there is minimal seasonal variation in sunlight availability throughout the year. However, recent research has demonstrated that vitamin D deficiency [2] remains prevalent in the general population, not only in high-latitude regions with marked seasonal fluctuations but also in low-latitude countries [3–6].

Although vitamin D deficiency has received greater attention in older populations [7], young individuals may also be at risk for this condition, even in low-latitude countries such as Brazil [8]. Furthermore, the lifestyle transition experienced by this age group may contribute to an increased risk of metabolic syndrome and cardiovascular diseases, which may be exacerbated by vitamin D deficiency [9].

The relationship between vitamin D status and biochemical markers, such as lipid, glycemic, and inflammatory profiles, has been studied in the general young population, however, it remains divergent [9]. For example, some studies have reported a significant association between vitamin D deficiency and higher levels of glucose, HOMA-IR, triglycerides, low-density lipoprotein (LDL), total cholesterol, and high-density lipoprotein (HDL) in adolescents and young adults [10–14]. Conversely, other published studies have found no relationship between vitamin D status and glucose, HDL, triglycerides, LDL, and cholesterol among adolescents [11, 13, 14]. This discrepancy may be due to variations in vitamin D thresholds used, population age, sex, and seasonal variations, which were not considered as influential factors in most published studies.

Several studies have indicated significant variations in 25(OH)D concentrations and the varying effects of vitamin D on disease outcomes among women and men [15–17]. This sex-related variation was believed to occur due to the difference in body fat mass content between the sexes [18]. However, recent analyses indicated that this difference might also be influenced by other factors, such as sun exposure habits, dietary intake, and different hormonal profiles between men and women [19]. Furthermore, these findings suggested that vitamin D supplementation should be considered sex-specific [19, 20]. Nevertheless, most studies investigating the impact of vitamin D on health have not considered sex as a crucial factor.

To date, only a few studies have evaluated the seasonal variations and sex-related differences in vitamin D status among healthy young adults living in low-latitude regions. Additionally, the effect of low vitamin D status on biochemical markers in this population remains unclear. Therefore, this cross-sectional study aimed to (a) analyze the serum concentrations of 25(OH)D in healthy young adults living in a low-latitude country during different seasons of the year; (b) determine the sex differences in serum 25(OH)D concentrations; and (c) investigate the association between vitamin D status and biochemical markers in young, healthy adults.

## Material and methods

### Study design and population

This cross-sectional study analyzed data (n = 203) from individuals recruited through an active search in a database of previous protocols and those who met the selection criteria were invited to participate in the present study, in addition to the invitation through social media. The inclusion criteria required young adults of both sexes, aged 18–24 years, and residing in the Federal District (latitude: 15°S), Brazil. Participants without serum 25(OH)D concentrations were excluded. The Federal District has a tropical savanna climate, with temperatures ranging from ~13°C to ~35°C throughout the year. In this region, the four seasons are not well defined, and there are mainly two seasons, the rainy season from October to March and the dry season from April to September. Data collection began in August 2019 but was suspended in March 2020 due to the COVID-19 pandemic. The study was conducted in accordance with the Helsinki Declaration guidelines and was approved by the Ethics Committee of the executing institution (CAAE:05185212.2.1001.5286). All participants were informed about the study and provided written consent. Authors did not have access to information that could identify individual participants during or after data collection.

### Demographic and biochemical parameters

Sociodemographic variables, including sex, age, and season of blood collection, were collected during blood sampling. Participants were instructed to fast for 10–12 hours prior to blood collection. Blood collection occurs during the Brazilian winter, spring, and summer months and each participant collects blood samples only once. All blood samples were stored refrigerated (0-10°C) until analysis. The following biomarkers were measured: total cholesterol, HDL-cholesterol, LDL-cholesterol, triglycerides, glycated hemoglobin (HbA1c), glucose, insulin, vitamin D, hs-CRP, parathyroid hormone (PTH), ionized calcium, and alkaline phosphatase. Cholesterol (mg/dL) was assessed by enzymatic kinetics, insulin (mU/L) by chemiluminescence, glucose (mg/dL) by the hexokinase method, HbA1c by ion-exchange chromatography, triglycerides (mg/dL) by kinetics, LDL-c and HDL-c by enzymatic colorimetric assay and intact PTH by electrochemiluminescence. Concentrations of 25(OH)D were assessed by chemiluminescence assay. The homeostasis model assessment index (HOMA-IR) was calculated using the following equation: [fasting insulin concentration (U/L) x fasting glucose concentration (mg/dL)] / 405, and β HOMA-beta (%) by = [20 X fasting insulin concentration (mU/L)] / [fasting glucose concentration (mmol/L) - 3.5]. Castelli’s risk index-I and II were calculated as total cholesterol (mg/dL) / HDL-c (mg/dL) and LDL-c (mg/dL) / HDL-c (mg/dL), respectively.

#### Statistical analysis

Variables are described as mean and standard deviation (SD). The Kolmogorov-Smirnov test assessed the normality distribution of variables. The independent variable 25(OH)D levels were categorized into the following thresholds: deficiency < 20 ng/mL; an insufficiency: 20 to 29.99 ng/mL and optimal ≥ 30 ng/mL in accordance with the US Endocrine Society [21] and the Position Statement Society for Adolescent Health and Medicine recommendation [22].

The study evaluated the distribution of variables of interest with respect to sex, vitamin D thresholds, and season. The association between sex and biochemical markers was analyzed using the Mann-Whitney test, while the Kruskal-Wallis test was used to assess the association between vitamin D thresholds and biochemical markers. The χ2-test was employed to determine the relationship between 25(OH)D concentration thresholds, sex, and season. Spearman correlation was used to explore the association between 25(OH)D status and biochemical markers. Standard linear regression models were utilized to investigate the predictive ability of vitamin D concentrations. The statistical analysis was performed using SPSS software for Windows (version 26.0, 2019; IBM Corp, Armonk, NY, USA), and statistical significance was defined as a p-value less than 0.05.

Post-hoc sample power was calculated using the G*Power software (version 3.1), based on the difference in serum 25(OH)D concentration between males (n = 71) and females (n = 132). We obtained a sample power of 81%, assuming the effect size of 0.429 and α error of 0.05.

## Results

A total of 259 individuals were invited to participate in the study, of those 56 were excluded due to the lack of 25(OH)D data. Overall, 203 participants were enrolled in the study, of whom 132 (65%) were women and 71 (35%) were men. The population characteristics and biochemical profile are presented in Table 1. The mean age of the participants was 21.17 ± 1.7 years, and there was no significant difference in age between women and men (p = 0.776). The majority of the participants (54.2%) provided blood samples during winter, followed by spring (40.4%), and only a small proportion during summer (5.4%). The winter season was the most frequent sampling period for men (50.7%) and women (61.4%) (p = 0.016).

**Table 1:** Characterization of the study population (n = 203).

Variables	Females (n = 132)	Males (n = 71)	Total (n = 203)	*p*-Value^†^
Age, years	21.19 ± 1.2	21.21 ± 1.8	21.17 ± 1.7	0.776
Seasons				
Winter	81 (73.6%)	29 (26.4%)	110 (54.2%)	0.016^††^
Spring	46 (56.1%)	36 (43.9%)	82 (40.4%)	
Summer	5 (45.5%)	6 (54.5%)	11 (5.4%)	
Total cholesterol, mg/dL	160.1 ± 30.1	157.39 ± 35.4	159.15 ± 32.0	0.368
Triglycerides, mg/dL	86.48 ± 11.3	81.96 ± 31.0	84.90 ± 39.6	0.950
LDL-c, mg/dL	91.33 ± 25.6	96.35 ± 33.5	93.08 ± 29.2	0.474
HDL-c, mg/dL	54.66 ± 11.3	48.30 ± 10.01	52.43 ± 11.2	<0.001
Non-HDL-, mg/dL	105.44 ± 27.8	109.10 ± 34.3	106.72 ± 30.2	0.642
Castelli risk index-I	3.14 ± 0.8	3.37 ± 0.9	3.016 ± 0.7	0.007
Castelli risk index-II	1.86 ± 0.7	2.1 ± 0.9	1.74 ± 0.6	0.005
Insulin, μUI/mL	10.15 ± 7.1	8.48 ± 4.8	9.56 ± 6.4	0.037
Blood Glucose, mg/dL	79.44 ± 6.8	81.04 ± 8.0	80.00 ± 0.5	0.020
HOMA-IR (n = 189)	2.09 ± 1.5	1.75 ± 1.0	1.98 ± 1.4	0.086
HOMA-β	231.59 ± 146.7	154.79 ± 67.8	205.58 ± 130.7	<0.001
HbA1c	5.15 ± 0.2	5.20 ± 0.3	5.17 ± 0.2	0.187
hs-CRP, mg/dL (n = 195)	0.37 ± 0.9	0.15 ± 0.2	0.29 ± 0.8	0.296
PTH, pg/mL	28.70 ± 9.1	27.58 ± 9.1	28.30 ± 9.1	0.226
Alkaline phosphatase, U/L	64.61 ± 17.5	79.77 ± 22.8	69.91 ± 20.8	<0.001
Ionized calcium, mmol/L	1.16 ± 0.04	1.15 ± 0.03	1.16 ± 0.04	0.026

Values are mean ± SD or n (%). High-density lipoprotein (HDL-c); Homeostasis model assessment (HOMA index) was calculated as fasting insulin (mU/L) × fasting glucose (mg/dL)/405. Castelli risk index-I and II was calculated as total cholesterol/HDL-c and LDL-c/HDL-c, respectively.

† Mann-Whitney test, unless said otherwise.

^**††**^ Chi-Square test.

Compared to men, women had higher mean concentrations of HDL-c (54.66 ± 11.3 mg/dL vs 48.30 ± 10.01 mg/dL; p<0.001), insulin (10.15 ± 7.1 μUI/mL vs 8.48 ± 4.8 μUI/mL; p = 0.037), and HOMA-β (231.59 ± 146.7 vs 154.79 ± 67.8; p<0.01) (Table 1). Conversely, men had a higher mean concentration of blood glucose than women (81.04 ± 8.0 mg/dL vs 79.44 ± 6.8 mg/dL; p = 0.020). Among all subjects, 11.6% presented insulin resistance according to HOMA-IR.

### Sex-related variation

**Fig 1:**
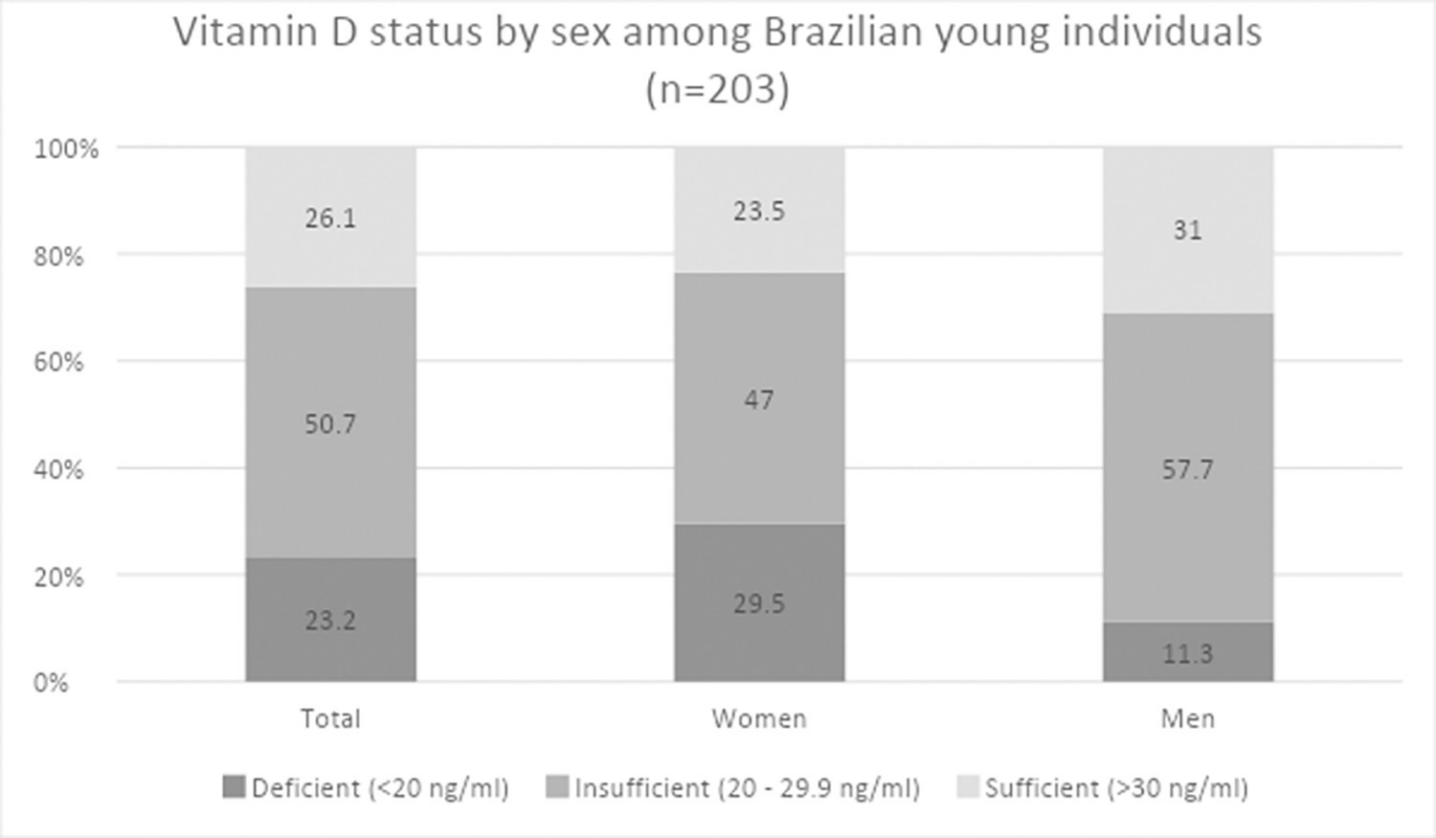
Vitamin D status by sex among young Brazilian individuals (n = 203). Statistical analysis: ^1^ Chi-square test between women and men.

Total mean vitamin D concentration was 25.76 ± 7.0 ng/mL. Men had higher mean serum vitamin D concentrations than women (27.70 ± 7.2 ng/mL vs 24.71 ± 6.7 ng/mL, p = 0.004). Only 26.1% of the sample had optimal vitamin D levels, with women having a lower prevalence than men (23.5% vs 31.0%) (Fig 1). Vitamin D insufficiency was observed in 50.7% of the sample, while 23.2% were vitamin D deficient. Vitamin D deficiency was more prevalent in women (29.5%) compared to men (11.3%) [χ^2^ (2) = 8,713; p = 0.013].

### Seasonal variance

There was a significant association between the 25(OH)D threshold and season, for both women (p < 0.045) and men (p < 0.011). The proportion of deficient women (41.3%) and insufficient men (72.2%) was higher in samples collected during spring compared to those collected during winter (23.5% for women and 34.5% for men) (Table 2).

**Table 2:** Serum concentrations and 25(OH)D status by season in Brazilian young women (n = 127) and men (n = 55).

	Females			Males		
	Winter (n = 81)	Spring (n = 46)	p-Value	Winter (n = 29)	Spring (n = 36)	p-Value
25(OH)D, ng/mL	25.7 ± 6.8	22.6 ± 5.4	0.011^1^	30.7 ± 7.2	25.1 ± 6.3	< 0.001^1^
Vitamin D Status						
<20 ng/mL	19 (23.5)	19 (41.3)	0.045^2^	2 (6.9)	6 (16.7)	< 0.001^2^
20–29.99 ng/mL	39 (48.1)	21 (45.7)		10 (34.5)	26 (72.2)	
≥ 30 ng/mL	23 (28.4)	6 (13.0)		17 (58.6)	4 (11.1)	

Values are expressed in mean ± SD or *n* (%). Participants with blood collection during summer were excluded from this analysis due to the small sample size for this subgroup.

† Mann-Whitney test, unless said otherwise.

^**††**^ Chi-Square test.

Moreover, the mean serum 25(OH)D concentration during winter was significantly higher than that during spring for both women (25.7 ± 6.8 ng/mL in winter vs 22.6 ± 5.4 ng/mL in spring, p = 0.002) and men (30.7 ± 7.2 ng/mL in winter vs 25.1 ± 6.3 ng/mL in spring, p = 0.001). In men, the mean serum 25(OH)D concentration during winter (30.7 ± 7.2 ng/mL) was above the sufficiency threshold of 30 ng/mL (Table 2).

### Vitamin D status and biochemical markers

Upon analysing the biochemical profile based on vitamin D status and sex, no significant association was observed between vitamin D thresholds and age or any of the biochemical markers for both women (see S1 Table). For women, there were no significant correlations between serum 25(OH)D concentrations and biochemical markers, except for a negative correlation with HOMA-β (r = -0.222; p = 0.012) when all women were included in the analysis. However, this correlation was not observed when only evaluating data from participants with vitamin D insufficiency and deficiency (Table 3).

**Table 3:** Correlation between serum 25(OH)D concentrations and biochemical markers in Brazilian young women and men (n = 132).

Biochemical Markers	25(OH)D							
	Females (n = 132)		Females with 25(OH)D insufficiency and deficiency, without control (n = 101)		Males (n = 71)		Males with 25(OH)D insufficiency and deficiency, without control (n = 49)	
	r	p	r	p	r	p	r	p
Blood Glucose, mg/dL	0.085	0.345	0.078	0.441	0.224	0.061	0.247	0.087
Insulin, μUI/mL	-0.099	0.260	-0.082	0.417	-0.013	0.915	-0.096	0.510
HOMA-IR	-0.083	0.342	-0.072	0.474	-0.026	0.831	0.009	0.950
HOMA-β (n = 128)	-0.222	0.012	-0.195	0.054	-0.210	0.093	-0.324	0.032
HbA1c	-0.039	0.660	-0.006	0.953	0.031	0.800	0.115	0.436
Total cholesterol, mg/dL	-0.023	0.793	-0.071	0.480	-0.252	0.034	-0.240	0.096
Triglycerides, mg/dL	-0.108	0.218	-0.117	0.244	-0.214	0.074	-0.415	0.003
HDL, mg/dL	-0.116	0.186	-0.078	0.439	0.071	0.556	0.147	0.314
LDL, mg/dL	0.090	0.304	0.010	0.918	-0.255	0.032	-0.256	0.076
Castelli’s risk index-I	0.102	0.246	0.018	0.854	-0.292	0.013	-0.279	0.053
Castelli’s risk index-II	0.132	0.131	0.024	0.814	-0.276	0.020	-0.284	0.048
hs-CRP, mg/dL	-0.151	0.085	-0.121	0.227	0.006	0.962	-0.102	0.491
Ionized calcium, mmol/L	0.026	0.771	-0.011	0.917	-0.113	0.349	0.089	0.541
Alkaline phosphatase, U/L	-0.114	0.194	-0.081	0.422	-0.070	0.561	0.080	0.587
PTH, pg/mL	-0.089	0.311	-0.132	0.189	0.132	0.273	-0.038	0.795

25(OH)D: 25 hydroxyvitamin D; High-density lipoprotein (HDL-c); HDL: high-density lipoprotein; LDL: low-density lipoprotein; hs-CRP: High-Sensitivity C-Reactive Protein; PTH: parathyroid hormone.

In men, the total sample showed an inverse correlation between vitamin D concentration and total cholesterol (r = -0.252; p = 0.034) and LDL (r = -0.235; p = 0.032) (Table 3). When analysing only participants with vitamin D insufficiency and deficiency (n = 49), an inverse correlation was found between 25(OH)D levels and HOMA-β (r = -0.234, p = 0.032) and triglycerides (r = -0.415, p = 0.003) (Table 3).

Linear regression models for men with vitamin D insufficiency and deficiency (n = 49) showed that vitamin D concentrations accounted for 10% of the total variance in HOMA- β (p = 0.029), with concentrations decreasing by 0.015 for each additional ng/ml of 25(OH)D, and 8% of the total variance in LDL (p = 0.041), with concentrations decreasing by 0.105 for each additional ng/ml of 25(OH)D. The model for total cholesterol did not reach statistical significance.

## Discussion

This study reveals a significant proportion (73.9%) of young individuals living in Brasília, Brazil with suboptimal vitamin D levels. The prevalence of this condition was higher among females and during the spring season. Among male participants with vitamin D insufficiency and deficiency, lower vitamin D concentrations correlated with elevated HOMA-β, triglycerides, and Castelli’s risk index-II. Our findings are consistent with previous reports of a high prevalence of hypovitaminosis D in young people residing in sunny countries [23, 24]. Despite the abundance of UVB radiation in tropical countries, which is higher than in high-latitude countries, lifestyle factors that affect sunlight exposure frequency and intensity may have contributed significantly to the observed high prevalence of hypovitaminosis D [25, 26].

Previous studies have reported lower vitamin D concentrations in females compared to age-matched males, as also observed in our study [19]. This difference in vitamin D status may be attributed to the higher subcutaneous fat content present in women [19]. Additionally, since serum 25(OH)D is a fat-soluble molecule, it can be stored in adipose tissue, leading to the sequestration of some circulating 25(OH)D from blood [27]. Other potential factors contributing to the observed sex-related difference in vitamin D concentration include the lower frequency of sunscreen use among males, which has been reported to be three times lower in boys, and the greater frequency of outdoor physical activity among males, allowing for increased endogenous production of vitamin D [24, 28]. However, a more comprehensive examination of the impact of sex on vitamin D status is necessary, and future studies should account for confounding variables such as seasonality and body fat percentage.

Our study revealed unexpected differences in vitamin D concentrations between seasons, with higher 25(OH)D levels observed in winter than in spring. This observation contradicts the assumption that colder seasons would lead to lower vitamin D status due to reduced sunlight exposure for endogenous production. However, it is worth noting that in low-latitude countries, such as Brazil, sunlight radiation is considered high throughout the year. During winter in Brazil, the UV Index may reach very high values (UV Index between 8 and 11) in practically all the North, Northeast, and Centre regions [29]. For example, Brasília, the city where the study was carried out, which is located in the Central region of Brazil. In contrast, in the Southeast of the country, medium and high UV Index are expected on clear sky days [30]. Additionally, mid-year school vacations in Brazil occur during the winter months (June-July), which may lead to increased sunlight exposure during recreational activities among young individuals. Thus, in low-latitude countries, the duration and frequency of sunlight exposure may be more influential than the season [21].

Another potential contributing factor to the unexpected differences in vitamin D concentrations between seasons in our study may be the typical climate in the study region. The rainy period occurs from November to April (mainly spring-summer), whereas from May to September (mainly autumn-winter), the region experiences drier conditions with fewer clouds [29]. This may lead to higher sunlight exposure during autumn and winter, as it would allow for more outdoor activities. Conversely, a higher presence of clouds and rain during the summer may reduce the production of endogenous vitamin D due to the reduced amount of ultraviolet B radiation that reaches the Earth’s surface and skin. Therefore, the unique combination of lifestyle habits and climate in Brazil may explain the higher levels of 25(OH)D observed during winter in our study.

Vitamin D and its metabolites have been shown to regulate lipid profiles through various mechanisms [28]. Recent discussions have drawn attention to the hypothesis that significant health outcomes may be associated with 25(OH)D concentrations only in cases of vitamin D deficiency and insufficiency, rather than in individuals with adequate vitamin D status [30, 31]. This is a crucial consideration for research in terms of sampling and data analysis. Consistent with previous observational studies [32, 33], our findings demonstrate an inverse correlation between serum 25(OH)D concentrations and triglyceride levels in individuals with vitamin D deficiency and insufficiency, although this association was observed only among young men. Higher vitamin D levels could provide a preventive effect by increasing intestinal calcium absorption, which decreases triglyceride synthesis in the liver [34]. Vitamin D also appears to play a crucial role in total cholesterol by increasing serum calcium levels, stimulating the secretion of bile salts, and reducing total cholesterol [34]. In this study, our analysis of all men revealed that higher 25(OH)D concentrations correlated with lower total cholesterol levels, providing further support for a potential protective role of vitamin D in regulating lipid profiles.

Recent studies have also investigated the association between serum vitamin D concentrations and glycemic regulation, as well as the onset of type II diabetes [35–37]. In a cross-sectional study of healthy Mexican young individuals, a negative correlation was found between vitamin D status and HOMA-IR and HOMA-β. When only individuals with deficient and insufficient levels of 25(OH)D were analyzed, those with vitamin D concentrations < 20 ng/mL were twice as likely to present insulin resistance (multivariate logistic regression OR = 2.1; 95% CI: 1.0, 9.5; p-trend 0.055) [11]. Lower 25(OH)D levels have also been linked to impaired glucose-stimulated insulin secretion and β-cell function, contributing to hyperglycemia in individuals with type 2 diabetes [37]. In our study, lower 25(OH)D concentrations were significantly correlated with higher HOMA-β in young men with inadequate vitamin D status, suggesting an increase in insulin secretory function. This result, accompanied by mean plasma glucose within the normal range in the groups with vitamin D insufficiency and deficiency, might indicate an early phase of insulin resistance [38, 39]. This stage is characterized by increased insulin levels as a compensatory mechanism to regulate plasma glucose concentrations [40]. Therefore, hyperglycemia manifests only when the function of pancreatic β-cells decreases [40]. Our findings suggest that lower serum vitamin D levels may be related to the early phase of insulin resistance in young men with inadequate vitamin D levels.

The current study had several strengths, including the collection of data in different seasons, allowing for the investigation of seasonal differences and the analysis of the relationship between vitamin D concentrations and various biochemical markers. However, there were also limitations, including the cross-sectional design, which precludes the establishment of causality between vitamin D status and the analysed biochemical parameters. Additionally, the lack of data on body composition, dietary and supplement intake, and sun exposure limited the discussion of the observed results. Another limitation is the non-representative sample, although all participants resided in the same city (latitude: 25°S) and were exposed to the same sunlight incidence.

In conclusion, our study found a high prevalence of vitamin D insufficiency and deficiency particularly in women and during the spring season and not in winter, which highlights the importance of evaluating weather and not just season. Our findings suggest that lower vitamin D levels may be related to higher levels of HOMA-β and triglycerides in young men, indicating a concern for insulin resistance and increased risk of cardiovascular disease in a population that is often overlooked in clinical evaluation and follow-up. Further studies with larger representative samples are needed to explore the mechanisms underlying the association between vitamin D and biochemical parameters.

## Supporting information

S1 Table Characteristics by vitamin D cut-off in Brazilian young women (n = 132) and men (n = 71). (DOCX)

S2 Table Linear regression models between serum 25(OH)D levels and biochemical markers in Brazilian young males and females. Model 1: all subjects, without adjusting. Model 2: only subjects with vitamin D insufficiency or deficiency, without adjusting.
(DOCX)
