# Supplementary material for: Seasonal and sex-related variation in vitamin D status and its association with other biochemical markers in young individuals: A cross-sectional study
Source: PLoS One. 2024 Mar 29;19(3):e0298862. doi: 10.1371/journal.pone.0298862 (PMC10980231; doi:10.1371/journal.pone.0298862)
Supplement: S1 Table — (DOCX) [file pone.0298862.s001.docx]

**S1 Table.** Characteristics by vitamin D cut-off in Brazilian young females (n= 132) and males (n=71).

| **Variables** | **Females** | | | | **Males** | | | |
| --- | --- | --- | --- | --- | --- | --- | --- | --- |
|  | **Deficiency**  **(n= 39)** | **Insufficiency**  **(n= 62)** | **Optimal**  **(n= 31)** | **p-Value** | **Deficiency**  **(n= 8)** | **Insufficiency**  **(n= 41)** | **Optimal**  **(n= 22)** | **p-Value** |
| Age, years | 21.1 ± 1.7 | 21.2 ± 1.5 | 21.3 ± 1.7 | 0.854 | 21.0 ± 1.7 | 21.4 ± 1.9 | 20.9 ± 1.8 | 0.432 |
| Glucose, mg/dL | 79.6 ± 8..0 | 79.0 ± 6.2 | 80.2 ± 6.8 | 0.835 | 78.2 ± 9.2 | 81.0 ± 9.0 | 82.0 ± 5.1 | 0.486 |
| Insulin, µUI/mL | 11.7 ± 7.9 | 9.0 ± 3.8 | 10.4 ± 10.2 | 0.250 | 7.8 ± 3.6 | 8.6 ± 5.3 | 8.4 ± 4.1 | 0.967 |
| HOMA-IR | 2.4 ± 1.8 | 1.7 ± 0.8 | 2.1 ± 2.1 | 0.485 | 1.6 ± 0.8 | 1.8 ± 1.2 | 1.7 ± 0.8 | 0.961 |
| HOMA-β (n=128) | 267.0 ± 143.7 | 224.2 ± 126.6 | 199.1 ± 108.9 | 0.077 | 202.3 ± 108.9 | 156.5 ± 72.6 | 147.5 ± 62.8 | 0.334 |
| HbA1c (%) | 5.1 ±0.2 | 5.2 ± 0.2 | 5.1 ± 0.2 | 0.903 | 5.1 ± 0.3 | 5.2 ± 0.3 | 5.2 ± 0.3 | 0.836 |
| Total cholesterol, mg/dL | 164.2 ± 34.4 | 158.1 ± 30.5 | 159.0 ± 22.7 | 0.939 | 180.9 ± 57.5 | 157.1 ± 31.2 | 149.4 ± 30.7 | 0.287 |
| Triglycerides, mg/dL | 102.1 ± 59.7 | 79.3 ± 29.3 | 91.2 ± 39.8 | 0.157 | 83.5 ± 21.2 | 80.8 ± 30.8 | 83.4 ± 35.3 | 0.732 |
| LDL-c, mg/dL | 81.2 ±30.8 | 89.5 ± 26.8 | 95.0 ± 19.9 | 0.416 | 123.7 ± 61.0 | 94.5 ± 27.5 | 89.8 ± 27.0 | 0.238 |
| HDL-c, mg/dL | 56.3 ± 14.3 | 55.0 ± 10.2 | 52.0 ± 8.5 | 0.463 | 46.4 ± 6.6 | 48.5 ± 10.6 | 48.6 ± 10.2 | 0.905 |
| hs-CRP, mg/dL | 0.6 ± 1.3 | 0.4 ±0.8 | 0.2 ± 0.3 | 0.356 | 0.2 ± 0.3 | 0.1 ± 0.3 | 0.2 ± 0.3 | 0.507 |
| IonisedIonized calcium, mmol/L | 1.2 ± 0.04 | 1.2 ± 0.04 | 1.2 ± 0.03 | 0.968 | 1.1 ± 0.01 | 1.1 ± 0.03 | 1.1 ± 0.03 | 0.228 |
| Alkaline phosphatase, U/L | 66.2 ± 15.1 | 65.1 ± 19.0 | 61.6 ± 17.5 | 0.487 | 85.4 ± 26.0 | 80.1 ± 21.0 | 77.0 ± 25.4 | 0.512 |
| PTH, pg/mL | 29.2 ± 9.5 | 28.4 ± 8.5 | 28.6 ± 9.8 | 0.936 | 29.6 ± 9.3 | 26.0 ± 9.7 | 29.7 ± 7.8 | 0.111 |

Values are mean ± SD. High-density lipoprotein (HDL-c); Homeostasis model assessment (HOMA index) was calculated as fasting insulin (mU/L) × fasting glucose (mg/dL)/405; **^†^**All analyses were done by Kruskal-Wallis test.
