# Supplementary material for: Seasonal and sex-related variation in vitamin D status and its association with other biochemical markers in young individuals: A cross-sectional study
Source: PLoS One. 2024 Mar 29;19(3):e0298862. doi: 10.1371/journal.pone.0298862 (PMC10980231; doi:10.1371/journal.pone.0298862)
Supplement: S2 Table — Model 1: all subjects, without adjusting. Model 2: only subjects with vitamin D insufficiency or deficiency, without adjusting. (DOCX) [file pone.0298862.s002.docx]

**S2 Table.** Linear regression models between serum 25(OH)D levels and biochemical markers in Brazilian young males and females.

| **Variables** | **Males** | | | | | | | | | | **Females** | | | | | | | | | |
| --- | --- | --- | --- | --- | --- | --- | --- | --- | --- | --- | --- | --- | --- | --- | --- | --- | --- | --- | --- | --- |
|  | **Model 1 (n=71)** | | | | | **Model 2 (n=49)** | | | | | **Model 1 (n=132)** | | | | | **Model 2 (n=101)** | | | | |
|  | **R^2^** | **B** | **β adjusted** | **t** | **P-Value** | **R^2^** | **B** | **β adjusted** | **t** | **P-Value** | **R^2^** | **B** | **β adjusted** | **t** | **P-Value** | **R^2^** | **B** | **β adjusted** | **t** | **P-Value** |
| HOMA-β | 0.039 | -0.019 | -0.198 | -1.603 | 0.114 | 0.1009 | -0.015 | -0.330 | -2.267 | **0.029** | 0.040 | -0.010 | -0.201 | -2.299 | **0.023** | 0.033 | -0.005 | -0.182 | -1.817 | 0.072 |
| Total cholesterol, mg/dL | 0.071 | -0.055 | -0.267 | -2.300 | **0.024** | 0.073 | -0.026 | -0.270 | -1.919 | 0.061 | 0.002 | -0.011 | -0.048 | -0.543 | 0.588 | 0.010 | -0.012 | -0.100 | -0.998 | 0.321 |
| Triglycerides, mg/dL | 0.026 | -0.037 | -0.161 | -1.353 | 0.181 | 0.135 | -0.045 | -0.368 | -2.712 | **0.009** | 0.012 | -0.017 | -0.109 | -1.256 | 0.212 | 0.026 | -0.014 | -0.163 | -1.639 | 0.104 |
| LDL-c, mg/dL | 0.068 | -0.056 | -0.261 | -2.246 | **0.028** | 0.100 | -0.032 | -0.317 | -2.291 | **0.026** | 0.003 | 0.015 | 0.059 | 0.672 | 0.502 | 0.000 | -0.003 | -0.021 | -0.206 | 0.837 |
| HDL-c, mg/dL | 0.001 | 0.020 | 0.028 | 0.232 | 0.817 | 0.017 | 0.046 | 0.130 | 0.898 | 0.374 | 0.021 | -0.086 | -0.144 | -1.665 | 0.098 | 0.014 | -0.039 | -0.117 | -1.174 | 0.243 |
| hs-CRP, mg/dL | 0.009 | 2.380 | 0.095 | 0.788 | 0.434 | 0.042 | -2.842 | -0.205 | -1.422 | 0.162 | 0.030 | -1.242 | -0.174 | -2.010 | **0.047** | 0.026 | -0.612 | -0.163 | -1.639 | 0.104 |
| Castelli`s Risk Index-I | 0.057 | -1.815 | -0.240 | -2.052 | **0.044** | 0.086 | -1.054 | -0.293 | -2.102 | **0.041** | 0.002 | 0.396 | 0.041 | 0.471 | 0.639 | 0.001 | -0.160 | -0.030 | -0.300 | 0.765 |
| Castelli`s Risk Index-II | 0.053 | -1.862 | -0.231 | -1.972 | **0.053** | 0.089 | -1.118 | -2.99 | -2.145 | **0.037** | 0.006 | 0.806 | 0.077 | 0.879 | 0.381 | 0.000 | -0.022 | -0.004 | -0.038 | 0.969 |

Model 1: all subjects, without adjusting. Model 2: only subjects with vitamin D insufficiency or deficiency, without adjusting.
